# Feasible and effective reuse of municipal sludge for vegetation restoration: physiochemical characteristics and microbial diversity

**DOI:** 10.1038/s41598-018-37338-4

**Published:** 2019-01-29

**Authors:** Xueya Liu, Lijuan Liu, Pingsheng Leng, Zenghui Hu

**Affiliations:** 1 Beijing Advanced Innovation Center for Tree Breeding by Molecular Design, Beijing University of Agriculture, Beijing, 102206 P.R. China; 2 College of Landscape Architecture, Beijing University of Agriculture, Beijing, 102206 P.R. China; 3 Beijing Collaborative Innovation Center for Eco-environmental Improvement with Forestry and Fruit Trees, Beijing, 102206 P.R. China; 4 Beijing Laboratory of Urban and Rural Ecological Environment, Beijing, 102206 P.R. China

## Abstract

The large volume of municipal sludge causes environmental problems in cities worldwide. In this study, municipal sludge, mixed with construction waste residue, was used as substrate to plant *Ailanthus altissima*. The growth of *A*. *altissima*, the substrate characteristics, and substrate microbial diversity were measured to investigate potential recycling and reusing pathways of municipal sludge. The obtained results showed that compared to garden soil, the mixed substrate was weakly alkaline, and had higher nutrient contents, which is beneficial for *A*. *altissima*, and results in better growth in mixed substrate. Although the contents of the main heavy metals in the mixed substrate were significantly higher than in garden soil, the values met the criterion of Class II soil in the Environmental Quality Standard for the Soils of China (GB15618-1995). Different substrates showed a variety of microbial diversities. *Proteobacteria* was the top microbial phylum in all samples, and higher relative abundances were found in samples containing municipal sludge. After growing *A*. *altissima*, the relative abundances of *Acidobacteria* and *Gemmatimonadetes* increased in the mixed substrate. Therefore, addition of construction waste residue and growth of *A*. *altissima* caused a difference. The microbial communities in the mixed substrate with *A*. *altissima* are both plant friendly and environmentally friendly. These results suggest this mixed substrate as a potentially feasible and effective pathway for the reuse and recycling of municipal sludge for vegetation restoration.

## Introduction

Excessive amounts of municipal sludge with high pollutant content have become a worldwide environmental problem. Especially in cities, the treatment and disposal of municipal sludge poses severe challenges due to land shortages, strict legal regulations, increasing costs, and public concern^1^. For example, China generates a large volume of sewage per year. In 2015 about 40 million tons of municipal sludge were generated, and the yield has been predicted to reach almost 80 million tons. Reducing the volume of municipal sludge is the dominant disposal goal of municipal sludge treatment. In China, landfill, composting, natural drying, and burning are the four main treatment modes of municipal sludge. The disposal amount by landfill accounts for 65.00% of the total municipal sludge, which may result in secondary pollution. Ecological and environmentally friendly recycling and reusing modes are urgently required for the treatment of municipal sludge.

Municipal sludge contains an abundance of nutrients. Dewatered municipal sludge contains 50.00–70.00% organic matter, 30.00–50.00% mineral components, 3.40–4.00% N, 0.50–2.50% P, and a large amount of micronutrients^2–4^. Therefore, municipal sludge has good potential for use as soil conditioner and fertilizer in land application. Due to relatively low contents of lignin or cellulose, the organic matter in municipal sludge mineralizes and degrades rapidly, thus generating and increasing abundant nitrate in soils^5^. The addition of municipal sludge composts to the soil improves soil properties, such as bulk density, porosity, and water holding capacity^6^. A significant enrichment in mineral nitrate and available phosphorus is detected in soil after municipal sludge application and both the pH value and electrical conductivity also increase^7^. However, municipal sludge also contains different contaminants, including heavy metals, polycyclic aromatic hydrocarbons (PAHs), polychlorinated biphenyls (PCBs), adsorbable organohalogens (AOX), pesticides, surfactants, hormones, pharmaceuticals, and nanoparticles^8^. These can create potential pollution to the soil and are hazardous to the health of humans^9,10^. Therefore, the land application of municipal sludge in agriculture and horticulture has security risks, while the application of municipal sludge in forests is a relatively ideal disposal pathway. The advantages are that forests are often deficient in major nutrients that can be found in abundance in municipal sludge, and that forests are not the major contributors to the human food chain.

Municipal sludge not only improves forestry soil fertility by increasing soil organic matter and available nitrogen and phosphorus, but also promotes the growth and reproduction of trees, including increases in the height and diameter of trees, which is advantageous for the growth of shrubs and herbs of trees^11^. The application of municipal sludge containing low heavy metal concentrations also improves the structure and the organic matter of the clay loam brown forest soil^12^. Moreover, municipal sludge has also been used as substrate for vegetation restoration and reconstruction^13,14^, which is a disposal route with broad prospect. All relevant previous studies have focused on the effect of municipal sludge on soil properties and plant growth; however, the influence on affected microbes received little attention. On the one hand, the many microbes in municipal sludge are pathogenic species, which may result in the pollution of soil. On the other hand, many microbes also can improve the physical and chemical features of soil to promote the growth of plants. So during the application of municipal sludge, the change of microbe diversity need to be investigated.

The construction waste is a main solid waste in city, which causes huge consumption of limited resources and leads to serious environment problem. In some areas all or part of construction waste is unlawfully deposited on land, or in natural drainages including water, contrary to regulations to protect human health, commerce and the environment. At present, landfill is the main disposal pathway of the construction waste, which increases the burden on landfill loading and operation, and the waste from sources such as solvents or chemicals can result in soil and water pollution. China is the largest producer of construction waste in the world. In 2015, totally 2,400 million tons of construction waste was produced in China, which accounts for 40% of the total amount of municipal waste. It is predicted that the amount of construction waste will arrived at 50 and 75 tons in 2020 and 2030, respectively^15^. But at present, the recycling utilization rate of construction waste is only about 5% in China, so the big city besieged by plenty of waste, which harms the urban safety and ecological development. Thus the effective and harmless method for the disposal of construction waste is in serious demand. Basis on the laboratory study of construction waste in the substrate mix for extensive green roofs^16^, we think that using construction waste as substrate in vegetation restoration is feasible.

In this study, the application of construction waste combined with municipal sludge in vegetation restoration was considered. The seeds of *Ailanthus altissima* were sowed on the substrate composed of municipal sludge mixed with construction waste residue. Then the growth of *A*. *altissima*, the substrate characteristics, and substrate microbial diversity were measured to investigate the potential recycling and reusing pathway of municipal sludge.

## Results

**Figure 1:**
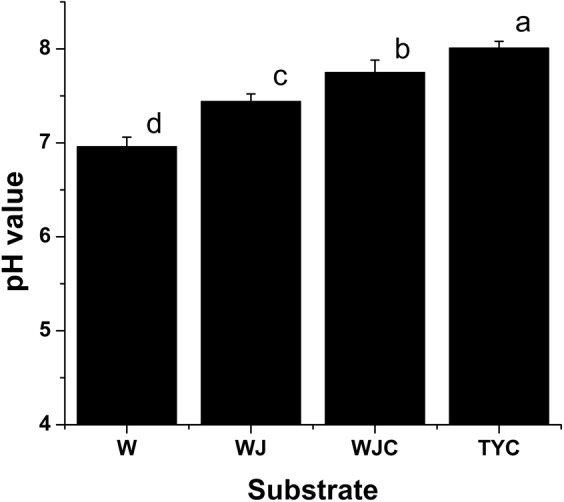
The pH values of substrates. Each bar is the average, and standard errors are shown. W, the pure sludge; WJ, the mixed substrate (sludge + construction waste) without planting *A*. *altissima*; WJC, the mixed substrate (sludge + construction waste) after planting *A*. *altissima*; TYC, the control substrate (garden soil) after planting *A*. *altissima*. Statistical significance [least significant difference (LSD)] of the difference in pH value of substrates is indicated by different small letters (*P* < 0.05).

The pH values of different substrates were tested (Fig. 1). The pH value of pure sludge (W) was about 7.00, while the pH value of garden soil (TYC) was about 8.00. When the sludge was mixed with construction waste residue (WJ), the pH value significantly increased by 6.90% compared with W (*P* <0.05). And after planting *A*. *altissima*, the pH value of mixed substrate (WJC) rose to 7.80.

Table 1 showed the nutrient contents of substrates. We found that in W, the nutrient contents were significantly higher than in TYC (*P* < 0.05). In W the contents of organic matter, total nitrogen, total phosphorus, and total potassium were 6.68, 11.23, 23.11, and 3.39 folds of those in TYC, respectively. Moreover, mixture with construction waste residue resulted in the significant decreases of these nutrient contents (*P* < 0.05), and further reduction was found after planting *A*. *altissima*. In WJC, the contents of organic matter, total nitrogen, total phosphorus, and total potassium decreased to 53.71%, 34.12%, 27.35%, and 45.86% of those in W, respectively, which was also lower than those in WJ; however, these values were still far higher than those in TYC (*P* < 0.05). The contents of available nitrogen, available phosphorus, and available potassium were also found to show similar patterns.

**Table 1:** The nutrient contents of substrates.

Nutrient index	Content (g·kg^−1^)			
	W	WJ	WJC	TYC
Organic matter	124.70 ± 2.34 a	77.06 ± 2.04 b	66.98 ± 0.80 c	18.68 ± 1.19 d
Total nitrogen	13.92 ± 0.36 a	5.5 ± 0.23 b	4.75 ± 0.10 c	1.24 ± 0.03 d
Available nitrogen	0.54 ± 0.01 a	0.51 ± 0.01 b	0.07 ± 0.00 c	0.07 ± 0.00 c
Total phosphorus	12.25 ± 0.75 a	4.51 ± 0.10 b	3.35 ± 0.07 c	0.53 ± 0.01 d
Available phosphorus	0.40 ± 0.01 b	0.43 ± 0.00 a	0.03 ± 0.00 c	0.03 ± 0.00 c
Total potassium	5.8 ± 0.17 a	4.23 ± 0.74 b	2.66 ± 0.04 c	1.71 ± 0.08 d
Available potassium	0.45 ± 0.01 a	0.45 ± 0.01 a	0.08 ± 0.00 b	0.09 ± 0.00 b

Note: Each value is the average, and standard errors are shown. Statistical significance [least significant difference (LSD)] of the difference in nutrient contents of substrates is indicated by different small letters (*P* < 0.05).

The contents of heavy metals in substrates were also examined (Table 2). It was found that the sludge-containing substrates had significantly higher contents of heavy metals than in TYC (*P* < 0.05), especially in W. The contents of Cu and Zn in W were 4.53 and 5.95-fold of those in TYC. Similar to the nutrient contents, the contents of heavy metals gradually decreased from W to WJ and to WJC. In WJC, the contents of As, Cd, Cr, Cu, Pb, and Zn were also significantly higher than those in TYC by 14.35%, 100.00%, 22.50%, 175.90%, 40.88%, and 317.88%, respectively (*P* < 0.05).

**Table 2:** Contents of heavy metals in substrates.

Heavy metal	Content (mg·kg^−1^)			
	W	WJ	WJC	TYC
As	18.14 ± 0.62 a	9.21 ± 0.67 b	7.33 ± 0.57 c	6.41 ± 0.25 d
Cd	0.71 ± 0.07 a	0.46 ± 0.04 b	0.38 ± 0.03 b	0.19 ± 0.01 c
Cr	53.73 ± 2.29 a	44.88 ± 0.72 b	32.72 ± 0.52 c	26.71 ± 0.83 d
Cu	98.56 ± 2.86 a	64.76 ± 1.74 b	59.98 ± 1.63 c	21.74 ± 0.62 d
Pb	26.56 ± 0.71 a	24.54 ± 0.79 b	23.06 ± 0.86 b	16.37 ± 0.41 c
Zn	347.12 ± 6.45 a	250.78 ± 4.47 b	243.71 ± 4.04 b	58.32 ± 2.54 c

Note: The average and standard errors are shown. Statistical significance [least significant difference (LSD)] of the difference in contents of heavy metals of substrates is indicated by different lower-case letters (*P* < 0.05).

**Figure 2:**
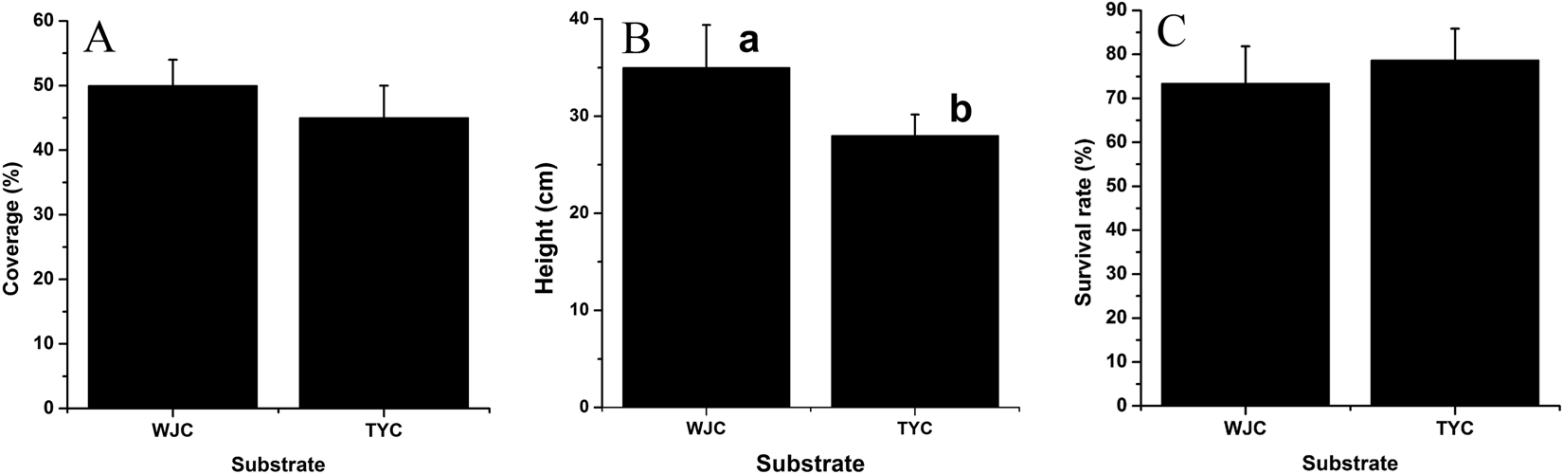
Survival rate (**A**), height (**B**), and coverage (**B**) of *A*. *altissima* in the WJC and TYC. Average values and standard errors are shown. Statistical significance [least significant difference (LSD)] of the difference in height of seedlings on substrates is indicated by different lower-case letters (*P* < 0.05).

After growth for eighteen months, the survival rate, height, and coverage of *A*. *altissima* were investigated (Fig. 2). The survival rate of in WJC was about 6.66% lower than that in TYC (Fig. 2A). However, the height and coverage of in WJC were higher than those in TYC (Fig. 2B,C). The height in WJC almost arrived at 35.00 cm, which was 25.00% higher than that in TYC. Compared to TYC, the coverage in WJC was 11.11% higher.

After high-throughput sequencing using the Illumina HiSeq platform, the data of samples were obtained and analyzed (Supplementary Table S1). The Q30 values of all samples exceeded 98.00%, and the GC values were about 55.00%. Moreover, Venn diagrams of OTUs exhibited the difference of microbial diversity between different samples (Supplementary Fig. 3).

**Figure 3:**
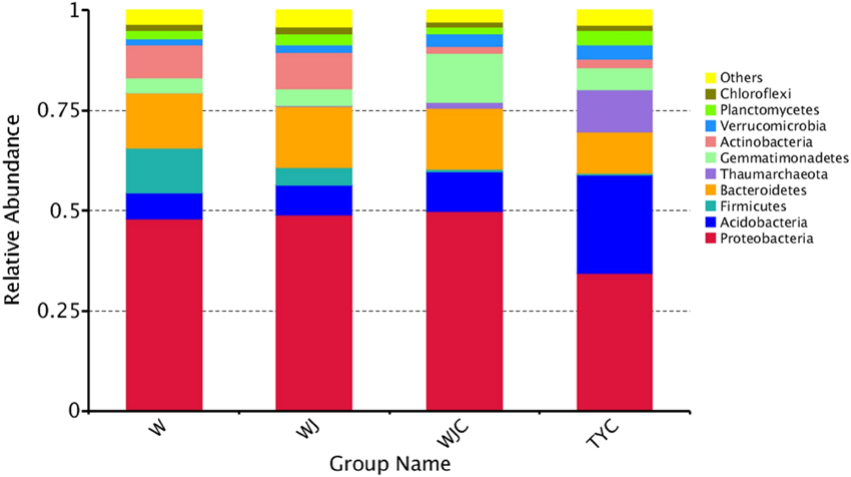
Relative abundance of the dominant microbial phyla observed in 16S rRNA gene sequences in different substrate samples.

To analyze the diversity among all samples, microbial species and their proportions at phylum levels are shown in Fig. 3. Due to the contained sludge, the dominant microbes showed similar proportions in W, WJ, and WJC, which largely differed from TYC. For these three samples, *Proteobacteria* was the largest proportion of population, which nearly arrived at 50.00%. In TYC, although *Proteobacteria* also occupied the highest proportion, it only reached about 35.00%, and *Acidobacteria* accounted for a further high proportion with about 25.00%. In W, WJ, and WJC, the proportion of *Acidobacteria* did not exceed 10.00%. *Bacteroidetes* also accounted for high proportions which was larger than that in TYC. *Acidobacteria* and *Thaumarchaeota* were found to gradually increase from W to TYC, while the proportion of *Firmicutes* presented the opposite pattern. In WJC and TYC *Gemmatimonacetes* also exhibited higher proportions than in W and WJ, which nearly arrived at 12.00% and 5.50%, respectively.

**Figure 4:**
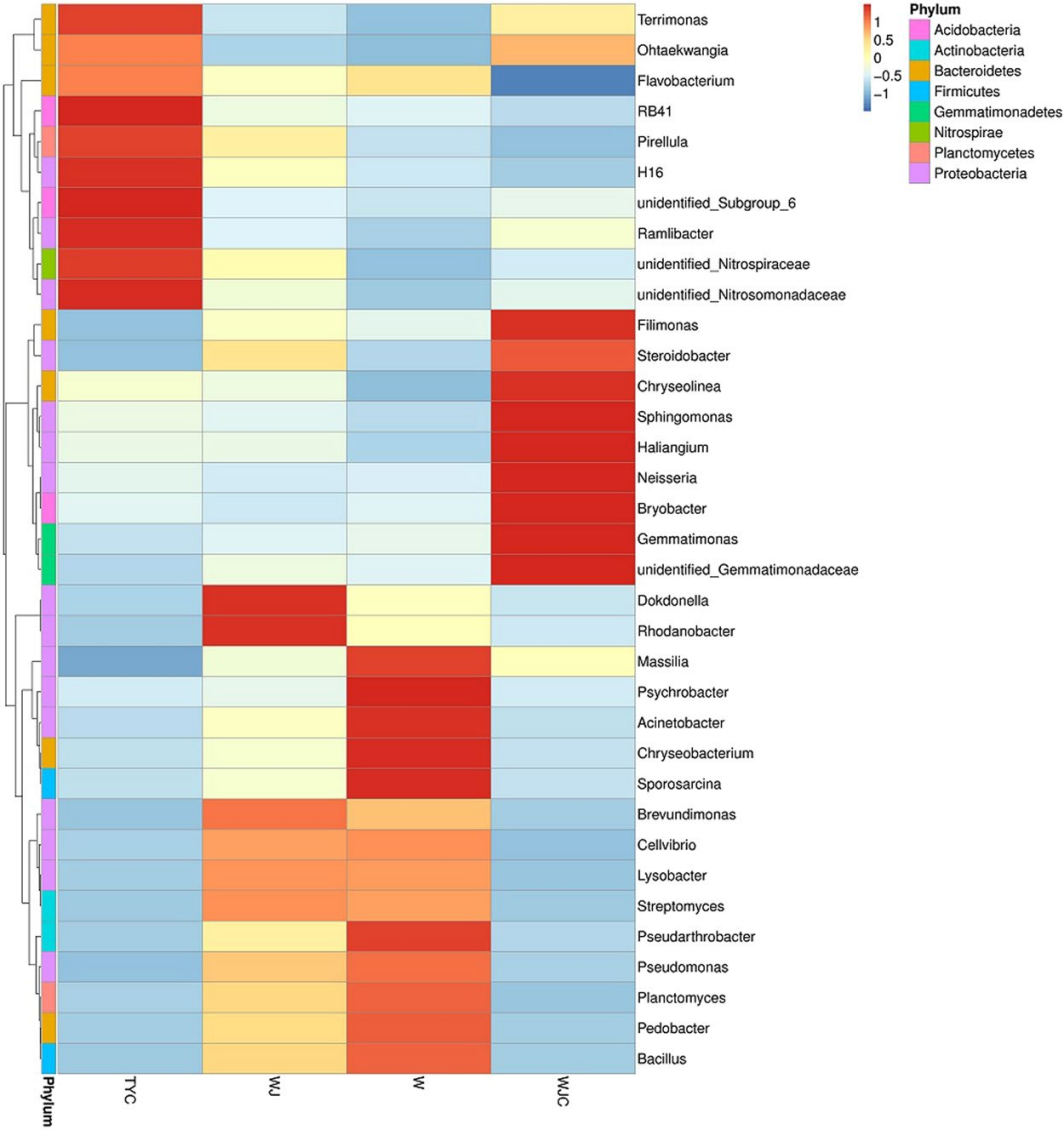
Hierarchically clustered heatmap analysis of microbial community at the genus level. The scale bar shows the standardized Z-value of the microbial relative percentages in four samples. The larger value in the scale bar shows the larger relative abundance of this genus in the sample.

To demonstrate the different compositions of the microbial community structures in four samples, hierarchical cluster analysis of microbial communities at the genus level in shown in Fig. 4. The relative abundances of these 35 genera varied significantly in different soil samples. However, in W and WJ, the relative abundances showed a similar pattern to some extent. Both in W and WJ, *Brevundimonas*, *Cellvibrio*, *Lysobacter*, *Streptomyces*, *Pseudomonas*, *Planctomyces*, *Pedobacter*, *Bacillus*, and *Pseudarthrobacter* presented relatively high abundances. In W, the abundances of *Massilia*, *Psychrobacter*, *Acinetobacter*, *Chryseobacterium*, *Sporosarcin*a, and *Pseudarthrobacter* were higher than those of other genera. In WJ, *Dokdonella* and *Rhodanobacter* exhibited the highest abundances. In WJC *Filimonas*, *Steroidobacter*, *Chryseolinea*, *Sphingomonas*, *Haliangium*, *Neisseria*, *Bryobacter*, *Gemmatimonas*, *unidentified_Gemmatimonadaceae*, and *Ohtaekwangia* were found to have high relative abundances. Ten genera (e.g., *Terrimonas*, *Ohtaekwangia*, *Flavobacterium*, *Pirellula*, and *Ramlibacter*) showed high abundances in TYC. In these samples, the microbial genera with high abundances belonged to more than two phyla, which indicated high microbial diversities in these samples.

**Figure 5:**
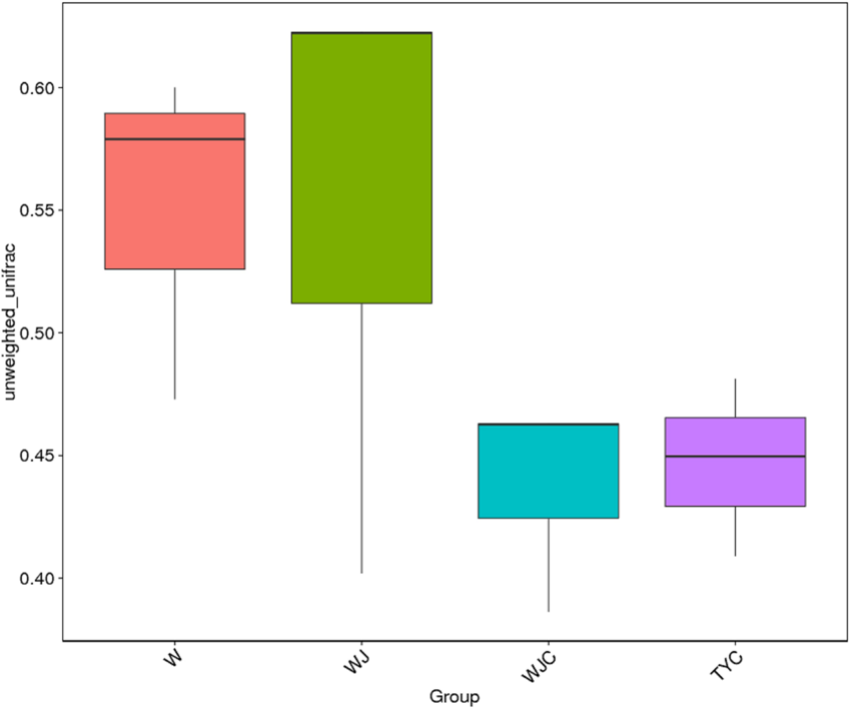
Difference analysis of beta diversity among four sample groups. The average and standard errors are shown. Turkey test was performed on the beta diversity statistical significance [least significant difference (LSD)] of the difference in contents of heavy metals of substrates is indicated by different lower-case letters (*P* < 0.05).

The beta diversity difference among samples of W, WJ, WJC, and TYC was analyzed in Fig. 5. Significant differences were not observed between W and WJ or between WJC and TYC. However, a significant difference was found between the samples (W and WJ) without plants and the samples (WJC and TYC) with planting *A*. *altissima*.

**Figure 6:**
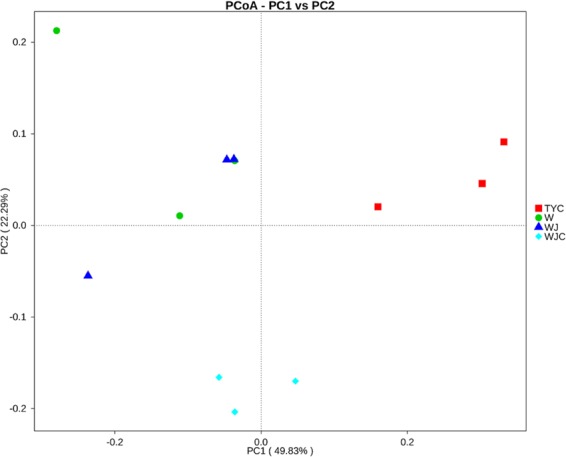
PCoA of microbial communities among different samples.

PCoA clearly showed variations of microbial communities among the four soil samples (Fig. 6). W and WJ were not separated and thus possessed similar microbial community compositions. WJC was separated from W and WJ along the second component (PC2), while TYC was separated from W and WJ along the first component (PC1).

**Figure 7:**
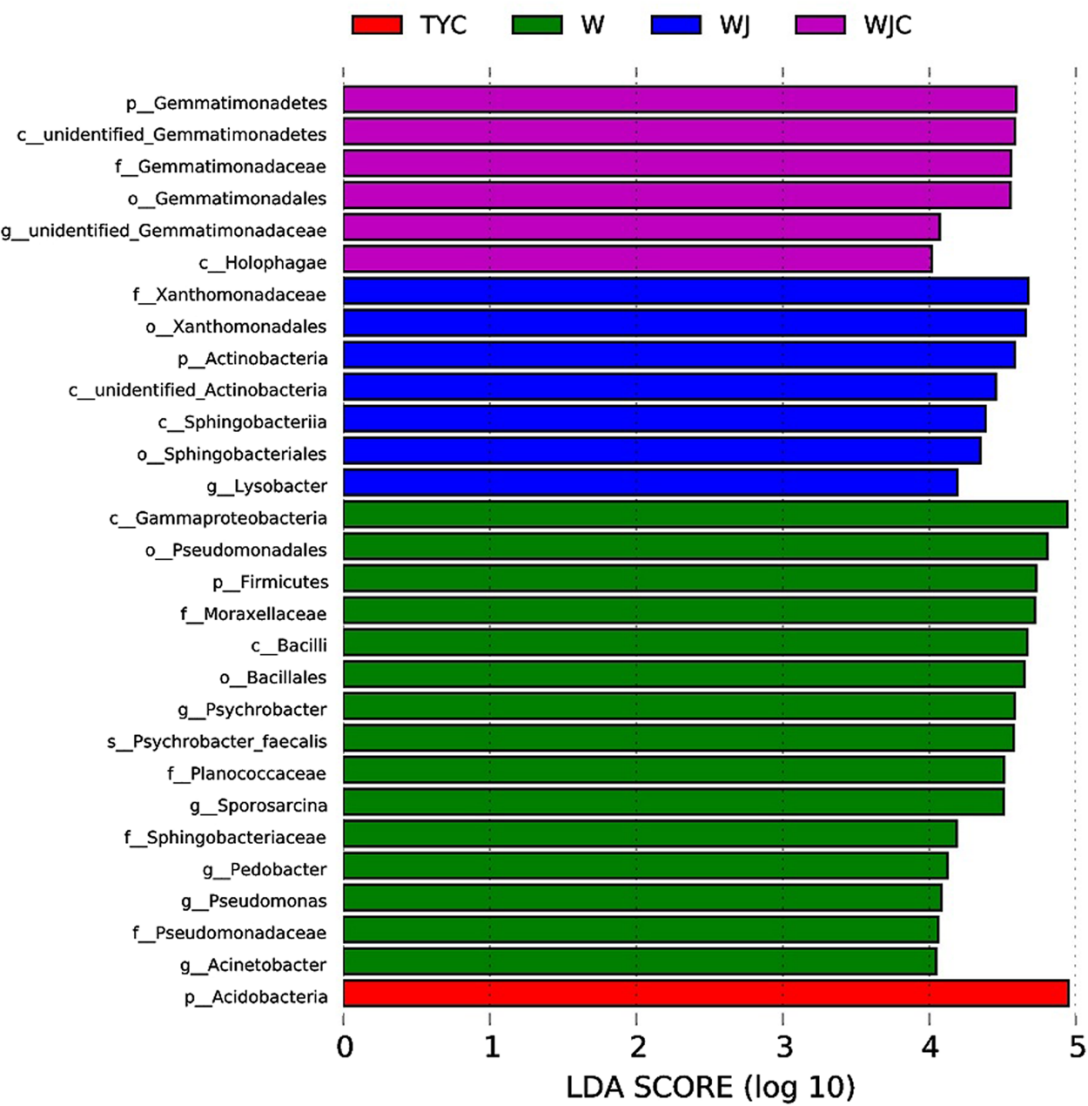
Liner discriminant analysis coupled with effect size measurements among different substrates. Lineages with LDA values higher than 4 are displayed.

A persistent difference in microbial taxa patterns was found via LEfSe analysis (Fig. 7). In W, the relative abundances of *Gammaproteobacteria* class, *Pseudomonadales* order, *Firmicutes* phylum, *Moraxellaceae* family, *Bacillales* order, *Psychrobacter* genus, *Psychrobacter* species, *Planococcaceae* family, *Sporosarcina* genus, Sphingobacteriaceae family, *Pedobacter* genus, *Pseudomonas* genus, *Pseudomonadacear* family, and *Acinetobacter* genus were higher than three other substrates, among which the *Gammaproteobacteria* class showed the highest LDA score. WJ had high relative abundances of *Xanthomooadaceae* family, *Xanthomonadeles* order, *Actinobacteria* pylum, *unidentified*_*Actinobacteria* class, *Sphingobacteriia* class, *Sphingobacteriales* order, and *Lysobacter* genus than other substrates. Compared to W, WJ, and TYC, in WJC *Gemmatimonadetes* phylum, *unidentified*_*Gemmatimonadetes* class, *Gemmatimonadaceae* family, *Gemmatimonadales* order, *unidentified*_*Gemmatimonadaceae* genus, and *Holophagae* class showed higher relative abundances. Only the phylum *Acidobacteria* was found to have higher relative abundance in TYC than the other three substrates.

## Discussion

Increasing concern is caused by the substantial generation of municipal sewage sludge and the inadequate development of the wastewater treatment industry, which causes environmental problems. Due to its high nutrient content, municipal sludge is used as soil conditioner and fertilizer to recycle its nutrients through the soil-plant system^17–19^. Sewage sludge has many beneficial effects on soil fertility, enriching it with its nitrogen, phosphorus, and micronutrient contents, and improving the physicochemical, microbiological, and enzymatic properties of soil^11,20,21^, which is an economic and effective method for disposal. However, a high level of contaminants, especially of heavy metals and pathogenic microbes, leads to a long-term environmental risk in soil^18^. Application of municipal sludge for vegetation restoration in a degraded ecosystem can avoid the potential risk that toxic contaminants enter the human food chain. This study investigated the potential application of municipal sludge combined with construction waste for vegetation restoration.

The utilized municipal sludge was found to have near neutral in pH, and the mixture with construction waste residue increased the pH value; moreover, after the growth of *A*. *altissima* for eighteen months, the pH value further increased. Although the mixed substrate was weakly alkaline, the pH value was evidently lower than that of garden soil. The application of sludge in tree peony-soil ecosystems also decreased the pH value, which was close to neutral soil pH, and was conducive to tree peony^20^. The municipal sludge used in this study had high nutrient contents (Table 1), which was too high for plants, while the construction waste residue contained few nutrients (Table 3). The addition of construction waste residue not only decreased the nutrient contents to a still high but acceptable level for plants (Table 1), it also improved the air permeability. *A*. *altissima* seedlings in the mixed substrate obtained better nutrient conditions than in garden soil; therefore, we can find that the height and coverage of *A*. *altissima* in mixed substrate was better than that in garden soil. The plant height, flower diameter, and flower numbers per plant of tree peony significantly increased after sludge compost amendment^22^. Application of sewage sludge compost as fertilizer in soil with mass ratios of 30.00% also promoted the height, ground diameter, and biomass of *Mangifera persiciforma* trees^23^. Compared to the application of sludge to soil, the mixed substrate of municipal sludge and construction waste residue also achieved the same beneficial effect of *A*. *altissima* plant growth; moreover, both of them were reused. Although the survival rate of *A*. *altissima* in mixed substrate was impacted, and was a little lower than in garden soil, it still achieved about 75.00%.

**Table 3:** Nutrient contents of construction waste residue.

Nutrient index	Content (g·kg^−1^)
Organic matter	0.68 ± 0.01
Total nitrogen	6.46 ± 0.03
Total phosphorus	0.22 ± 0.00
Total potassium	0.96 ± 0.01

Note: Average values and standard errors are shown.

**Figure 8:**
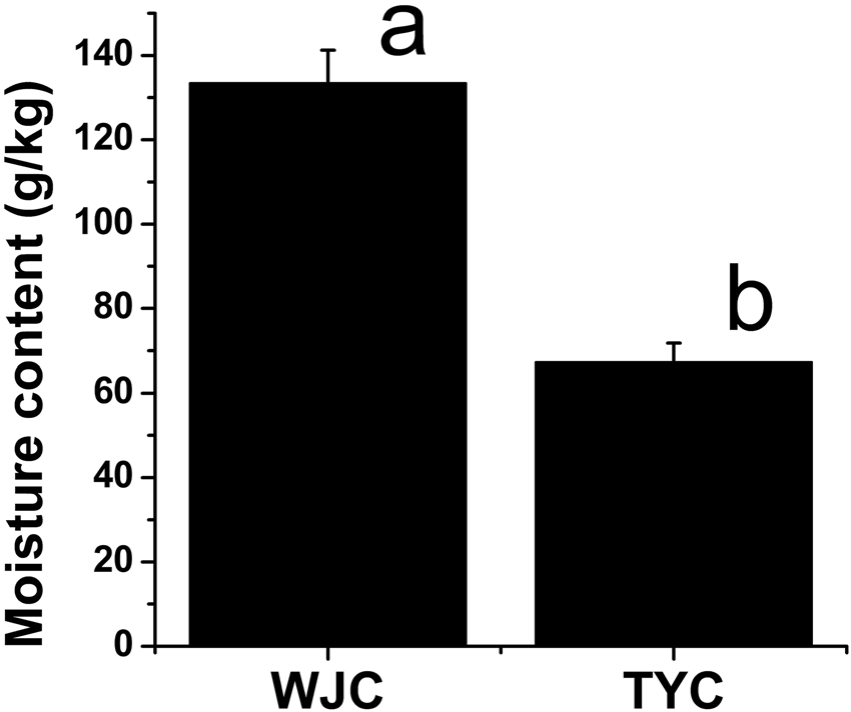
Moisture contents of WJC and TYC. The averages and standard errors are shown. Statistical significance [least significant difference (LSD)] of the difference in moisture contents of substrates is indicated by different lower-case letters (*P* < 0.05).

Moreover, after mixing with construction waste residue, the contents of main heavy metals in the municipal sludge decreased significantly. In the mixed substrate with *A*. *altissima* (with reference to Environmental Quality Standard for Soils of China, GB15618-1995), the contents of As, Cr, and Pb reached the level of first-degree soil, and the contents of Cd, Zn, and Cu met the criterion of second-degree soil. Many previous studies focused on the heavy metals during the land application of municipal sludge, and found that as long as the application amount was controlled within reasonable levels, the pollution and hazard of heavy metals could be avoided^7,23–26^. Based on our data, with regard to heavy metal contents, the mixed substrate belonged to Class II soil, which could be used to grow crops, vegetables, tea trees, fruit trees, and forage grasses, and would not cause pollution or be hazardous for plants and the environment. In addition, the moisture content (Fig. 8) and the activities of soil enzymes including urease, catalase, invertase, and alkaline phosphatase (Table 4) in the mixed substrate were significantly higher than those in garden soil, which was also beneficial for *A*. *altissima* growth.

**Table 4:** Activities of soil enzymes in WJC and TYC.

Soil enzymes	Activities	
	WJC	TYC
Urease (μg·g^−1^·2 h^−1^)	22.51 ± 1.72 a	4.76 ± 0.57 b
Catalase (mL·g^−1^·h^−1^)	1.43 ± 0.02 a	0.73 ± 0.02 b
Invertase (mg·g^−1^·24 h^−1^)	22.49 ± 0.39 a	9.96 ± 0.14 b
Alkaline phosphatase (μg·g^−1^·h^−1^)	448.34 ± 9.02 a	171.38 ± 6.77 a

Note: Average values and standard errors are shown. Statistical significance [least significant difference (LSD)] of the difference in soil enzyme activities of substrates is indicated by different lower-case letters (*P* < 0.05).

The microbes play an important role for the physicochemical properties of soil and plant growth. Large numbers of microbes can be found in municipal sludge, many of which are species that decompose organic matter, while some of them are pathogenic species that may be environmentally hazardous. Therefore, in the application of municipal sludge, the diversity of microbes is a focal point. In this study, using high-throughput sequencing on the Illumina HiSeq platform, the microbial diversity of mixed substrate was investigated. Our experimental results demonstrated that the microbial community structure observed in the substrate samples containing municipal sludge significantly differed from that of garden soil. Phylum abundance analysis showed that although *Proteobacteria* was the top microbial phylum in all samples, compared to garden soil, higher values were found in samples that contained municipal sludge. Furthermore, the relative abundance of *Proteobacteria* in activated sludge of a municipal wastewater treatment plant of Northern China in summer arrived at about 50.50%^27^. *Proteobacteria* included many important ammonia oxidizing bacteria involving nitrogen fixation, such as *Betaproteobacteria* and *Gammaproteobacteria*^28^. Therefore, municipal sludge can provide more nitrogen than garden soil. *Bacteroidetes*, *Fimicutes*, and *Actinobacteria* were the other main phyla in samples containing municipal sludge with higher relative abundance than garden soil. However, in a recent study, the proportions of *Fimicutes* and *Actinobacteria* in activated sludge were low^27^. This may result from differences in sludge sources. In garden soil, *Acidobacteria* and *Thaumarchaeota* were found to have a higher relative abundance compared to samples containing municipal sludge. *Acidobacteria* has been reported to be a main bacterial phylum in agricultural or other types of soils, with some variation in their relative abundance^29,30^. *Thaumarchaeota* was named by Brochier-Armanet *et al*.^31^, and plays an important role in the ammonia oxidization and carbon fixation in soil. No reports exist about *Thaumarchaeota* in municipal sludge. Therefore, a large difference was found between the substrate samples containing municipal sludge and garden soil.

In addition, after growing *A*. *altissima*, the relative abundances of *Acidobacteria* and *Gemmatimonadetes* increased in the mixed substrate, especially for *Gemmatimonadetes* whose relative abundance in WJC was also far higher than that in TYC. Moreover, the relative abundance of *Fimicutes* decreased after growing *A*. *altissima* in the mixed substrate. Therefore, it was demonstrated that the growth of *A*. *altissima* resulted in these microbial changes. The planting of apple seedlings was reported to exert a large impact on the bacterial community in the soils of replanted new planting sites^32^.

At the genus level, heat map analyses and PCoA also showed the difference among these four substrates. Similarly, at phylum level, the microbial communities in TYC were significantly different from W, WJ, and WJC, and compared to W and WJ, WJC was somewhat closer to TYC, which was also demonstrated by the analysis of beta diversity. As the dominant microbial communities in WJ, *Dokdonella* and *Rhodanobacter* presented low relative abundance in WJC. *Rhodanobacter* was reported to be likely involved in the denitrifying process, leading to denitrification in low pH soil^33^. In this study, *Rhodanobacter* was found to be part of the prominent microbial communities in the alkaline substrate. For common soil, *Rhodanobacter* had a negative effect on nitrogen nutrition supply due to nitrogen losses, while for the substrate containing municipal sludge with high nitrogen content, its denitrification can decrease the nitrogen nutrition content to a suitable level for the growth of *A*. *altissima*, which played a positive role. However, in WJC, nine microbial communities, such as *Filimonas*, *Chryseolinea*, *Sphingomonas*, and *Gemmatimonas* accounted for high relative percentages. It has been reported that *Sphingomonas* can effectively degrade dibenzo-p-dioxin^34^, hexachlorocyclohexane^35^, and phenazine-1-carboxylic acid^36^ in soils. Moreover, *Sphingomonas* was also capable to degrade fenvalerate, permethrin, fenpropathrin, beta-cypermethrin, cyhalothrin, deltamethrin, bifenthrin, and 3-PBA, and showed multi-resistance to heavy metals and antibiotics^37^. Therefore, *Sphingomonas* had potential for degrading organic pollutants, especially pesticides in substrate. Furthermore, *Gemmatimonas* was confirmed to reduce the emission of N_2_O, which is a potent greenhouse gas and ozone depletion agent originating from soil^38^. The high relative abundance of *Gemmatimonadaceae* suggested that WJC may be beneficial for the atmospheric environment.

## Conclusions

This study showed that using municipal sludge mixed with construction waste residue as substrate, increased the growth of *A*. *altissima* seedlings and the physiochemical characteristics were suitable for plants, suggesting that the substrates showed potential for vegetation restoration. The different substrates showed various microbial diversities. *Proteobacteria* was the top microbial phylum in all samples and a higher relative abundance were found in the samples containing municipal sludge. After growing *A*. *altissima*, the relative abundances of *Acidobacteria* and *Gemmatimonadetes* increased in the mixed substrate. Therefore, the addition of construction waste residue and the growth of *A*. *altissima* caused the difference. The microbial communities in WJC was plant-friendly and environment-friendly. The results suggested that using as substrate for vegetation restoration was a potentially feasible and effective pathway for the reuse and recycling of municipal sludge. The heavy metal contents in WJC met the criterion of the Class II soil although the values were significantly higher than in TYC. Therefore, using this mixture as substrate in vegetation restoration was a potentially feasible and effective pathway for the reuse and recycling of municipal sludge and construction waste residue.

## Materials and Methods

### Experimental Design

The experiment was conducted in the Science and Technology Park of the Beijing University of Agriculture. The municipal sludge used in this study was taken from the Xiaotangshan sewage treatment plant of the Changping district in Beijing, and was naturally dried in the air. The construction waste residue originated from a construction site near the Tingzizhuang village of Changping district in Beijing, and big bricks and stones were removed. The municipal sludge was mixed with the construction waste residue at a volume ratio of 1:1 to obtain the treatment substrate. The garden soil in the nursery was used as control substrate. Both types of experiment plots (5 m × 5 m) were paved with treatment and control substrate to 50 cm thickness, respectively. Three plots were prepared for every type, acting as three replicates.

*A*. *altissima* was used as plant material. Five hundred *A*. *altissima* seeds (China National Tree Seed Corporation, Beijing) were uniformly sowed (100 seeds per square meter) on two experimental plots in April of 2015. The *A*. *altissima* seedlings grew under natural condition.

### Soil Sampling

Pure sludge and mixed substrate without planting *A*. *altissima* were sampled. After planting *A*. *altissima*, both mixed and control substrates were collected in October of 2016. In each plot, at a depth of 10–15 cm, the substrates close to the roots of 10 *A*. *altissima* seedlings with medium size were collected and were divided into two parts. One part of the substrates was used to determine the characteristics, while the other part was frozen in liquid N_2_ immediately for subsequent DNA extraction. The samples are listed in Table 5.

**Table 5:** Collected samples.

Samples	Meaning
W	Pure sludge
WJ	Mixed substrate without planting *A*. *altissima*
WJC	Mixed substrate after planting *A*. *altissima*
TJC	Control substrate after planting *A*. *altissima*

### Substrate Characteristics Measurement

The pH value was determined with a Delta 320 pH meter (Mettler-Toledo International Inc., Switzerland) at substrate: water = 1:5. The content of organic matter was determined using the potassium dichromate volumetric method. Using the Kjeldahl determination, the content of total nitrogen was investigated in substrate after heating digestion by concentrated sulfuric acid. The contents of total phosphorus, total potassium, and heavy metals were determined by ICP-AES (6,300, Thermo Fisher Scientific Inc., USA) after microwave digestion.

### Growth Investigation of *A*. *altissima*

In October, the survival rate of *A*. *altissima* was calculated. The height of 30 seedlings with medium size were measured with a meter stick. Moreover, the coverage was investigated using a canopy projection method.

### DNA Extraction

Total genomic DNA from soil samples was extracted using the CTAB/SDS method. DNA concentration and purity were monitored on 1.00% agarose gels. Each sample extraction was conducted three times. DNA concentration and purity were monitored on 1.00% agarose gels. According to the concentration, DNA was diluted to 1 ng·L^−1^ using sterile water. Then, the DNA extractions were used as template DNA for high-throughput sequencing (HTS) analysis by Novogene Bioinformatics Technology Co., Ltd., Beijing, China.

### Microbial 16S rRNA Gene Amplification

The protocol of Caporaso *et al*.^39^ was used to determine the diversity and composition of the microbial communities in soil samples. PCR amplifications were conducted with the 515F-806R primer set amplifying the V4 region of the 16 S rDNA gene. All PCR reactions were conducted in 30 μL reactions with 15 μL of Phusion® High-Fidelity PCR Master Mix (New England Biolabs); 0.2 μM of forward and reverse primers, and about 10 ng template DNA. Thermal cycling consisted of an initial denaturation at 98 °C for 1 min, followed by 30 cycles of denaturation at 98 °C for 10 s, annealing at 50 °C for 30 s, and elongation at 72 °C for 30 s. Finally, 72 °C for 5 min.

### PCR Product Quantification and Qualification

The same volume of 1X loading buffer (contained SYB green) was mixed with PCR products and electrophoresis was conducted on a 2.00% agarose gel for detection. Samples with bright main strip between 400–450 bp were chosen for further experiments.

### PCR Products Mixing and Purification

PCR products were mixed at equidensity ratios. Then, mixture PCR products were purified with the GeneJET Gel Extraction Kit (Thermo Scientific).

### Library Preparation and Sequencing

Sequencing libraries were generated using the TruSeq DNA PCR-free Library Prep Kit for Illumina (NEB, USA) following manufacturer’s recommendations and index codes were added. The library quality was assessed on the Qubit@2.0 Fluorometer (Thermo Scientific) and Agilent Bioanalyzer 2100 system. Finally, the library was sequenced on an Illumina HiSeq platform and 250 bp paired-end reads were generated.

### Data Analysis

Paired-end reads from the original DNA fragments were merged using FLASH^40^, which is a very fast and accurate analysis tool that was designed to merge paired-end reads when there were overlaps between reads1 and reads2. Paired-end reads were assigned to each sample according to unique barcodes. Sequences were analyzed using the QIIME software package (Quantitative Insights Into Microbial Ecology)^39^, and in-house Perl scripts were used to analyze alpha- (within samples) and beta- (among samples) diversity. First, reads were filtered by QIIME quality filters. Then, pick_de_novo_otus.py was used to pick operational taxonomic units (OTUs) by making a OTU table. Sequences with ≥97.00% similarity were assigned to the same OTUs. A representative sequence was picked for each OTU and the RDP classifier was used to annotate taxonomic information for each representative sequence^41^. QIIME calculates unweighted unifrac, which is phylogenetic measure of beta diversity. Unweighted unifrac was used for Principal Coordinate Analysis (PCoA). To further mine the data of microbial diversity of the differences between the samples, LEfSe was conducted.

All experimental data were analyzed and using SPSS16.0. Statistical significance [least significant difference (LSD)] was performed on pH value, nutrient content, heavy metal content, survival rate, plant height, coverage, moisture content, and soil enzyme activity. Turkey tests were performed on the beta diversity. Origin 8.0 was used for plotting and the data in the figures are shown as the mean ± standard error.

## Supplementary information

Supplementary Table S1 and Figure S1
