# Supplementary material for: Feasible and effective reuse of municipal sludge for vegetation restoration: physiochemical characteristics and microbial diversity
Source: Sci Rep. 2019 Jan 29;9:879. doi: 10.1038/s41598-018-37338-4 (PMC6351607; doi:10.1038/s41598-018-37338-4)
Supplement: Supplementary file 1 — Supplementary Table S1 and Figure S1 [file 41598_2018_37338_MOESM1_ESM.pdf]

# Feasible and effective reuse of municipal sludge for vegetation restoration: physiochemical characteristics and microbial diversity

Xueya Liu[1,2], Lijuan Liu[1,2], Pingsheng Leng[1,2,3,*] & Zenghui Hu[1,2,3,4,*]

[1]Beijing Advanced Innovation Center for Tree Breeding by Molecular Design, Beijing University of Agriculture, Beijing, 102206 PR China
[2]College of Landscape Architecture, Beijing University of Agriculture, Beijing, 102206 PR China
[3]Beijing Collaborative Innovation Center for Eco-environmental Improvement with Forestry and Fruit Trees, Beijing, 102206 PR China
[4]Beijing Laboratory of Urban and Rural Ecological Environment, Beijing, 102206 PR China
Corresponding author: Pingsheng Leng, email: bualengpingsheng@163.com, Zenghui Hu, buahuzenghui@163.com.

**Supplementary Table S1**. Source data for analysis of microbial communities

| Samples | Raw PE | Raw Tags | Clean Tags | Total Tags | Taxon tags | Unique tags | OTUs | Q30% | GC% |
|---|---|---|---|---|---|---|---|---|---|
| W | 33,665 | 33,141 | 32,728 | 31,433 | 29,358 | 2,074 | 2,565 | 98.95 | 53.11 |
| WJ | 31,820 | 31,071 | 30,604 | 29,338 | 26,985 | 2,352 | 2,349 | 98.92 | 54.14 |
| WJC | 67,908 | 67,409 | 66,929 | 65,053 | 61,527 | 3,526 | 3,715 | 98.94 | 55.45 |
| TYC | 60,999 | 60,647 | 60,188 | 59,493 | 55,902 | 3,591 | 3,591 | 98.88 | 55.81 |

After high-throughput sequencing using the Illumina HiSeq platform, the data of samples were obtained and analyzed (Supplementary Table S1). The Q30 values of all samples exceeded 98.00 %, and the GC values were about 55.00 %. The samples of WJC and TYC presented higher tag amounts than W and WJ. The taxon tag amounts of WJC and TYC exceeded 55,000 and the amounts of W and WJ remained below 30,000. Every OTU presented one type of microbial species. The OTU amounts of WJC and TYC were close and exceeded 3,500. The OTU amounts of W and WJ were only 2,565 and 2,349, respectively, which were far lower than those of WJC and TYC.

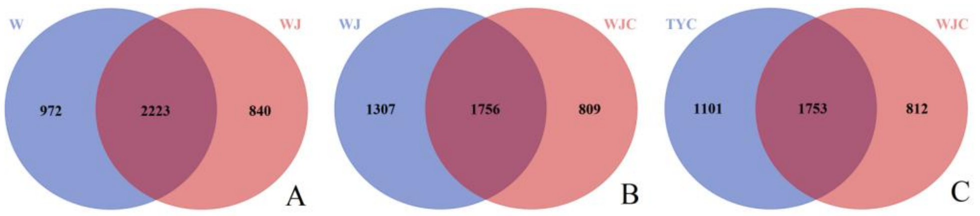


**Supplementary Figure S1**. Venn diagrams of OTUs.

Venn diagrams of OTUs are presented in Supplementary Fig. S1. 2,223 similar OTUs were found between W and WJ. The unique OTUs in W and WJ were 972 and 840, respectively. 1,756 similar OTUs were found between WJ and WJC, and the unique OTUs were 1,307 and 809, respectively. For samples after planting *A. altissima*, the similar OTUs were 748. The unique OTUs in WJC and TYC were 812 and 1,101, respectively. The difference of microbial diversity between WJ and WJC and between WJC and TYC was more apparent than between W and WJ.
